# Fully-Differential TPoS Resonators Based on Dual Interdigital Electrodes for Feedthrough Suppression

**DOI:** 10.3390/mi11020119

**Published:** 2020-01-21

**Authors:** Yi Zhang, Jing-Fu Bao, Xin-Yi Li, Xin Zhou, Zhao-Hui Wu, Xiao-Sheng Zhang

**Affiliations:** School of Electronic Science and Engineering, University of Electronic Science and Technology of China, Chengdu 611731, China; rubywing_1984@hotmail.com (Y.Z.); kimilee56@gmail.com (X.-Y.L.); xzhou@std.uestc.edu.cn (X.Z.); zhwu@std.uestc.edu.cn (Z.-H.W.)

**Keywords:** MEMS, resonator, TPoS, feedthrough, differential-in, differential-out

## Abstract

As one of the core components of MEMS (i.e., micro-electro-mechanical systems), thin-film piezoelectric-on-silicon (TPoS) resonators experienced a blooming development in the past decades due to unique features such as a remarkable capability of integration for attractive applications of system-on-chip integrated timing references. However, the parasitic capacitive feedthrough poses a great challenge to electrical detection of resonance in a microscale silicon-based mechanical resonator. Herein, a fully-differential configuration of a TPoS MEMS resonator based on a novel structural design of dual interdigital electrodes is proposed to eliminate the negative effect of feedthrough. The fundamental principle of feedthrough suppression was comprehensively investigated by using FEA (i.e., finite-element analysis) modeling and electrical measurements of fabricated devices. It was shown that with the help of fully-differential configuration, the key parameter of SBR (i.e., signal-to-background ratio) was significantly enhanced by greatly suppressing the in-phase signal. The *S*-parameter measurement results further verified the effectiveness of this novel feedthrough suppression strategy, and the insertion loss and SBR of proposed TPoS resonators were improved to 4.27 dB and 42.47 dB, respectively.

## 1. Introduction

Along with the development of wireless and portable electronic systems, the need to shrink the size of the systems is on the rise. Micro-electro-mechanical systems (MEMS) resonators with small form factor and potential integration capabilities with standard CMOS integrated circuits have been recognized as a promising alternative to replace quartz crystal resonators. As a keystone for the realization of the Internet of Things (IoT), these MEMS resonator devices have been extensively developed for implementation in a wide range of application fields due to a much higher quality factor (*Q*)—compared to electrical resonators (e.g., *LC* tank circuits)—smaller size, and lower power cost. These applications have included frequency synthesis and frequency selection in modern portable wireless communication systems, as well as a whole host of sensors that are based on the principle of modulating mechanical resonance [1,2,3,4,5,6,7,8,9].

But typically there is large parasitic feedthrough in MEMS resonators where the input drive signal is directly coupled to the output ports through parasitic elements. When reducing the physical dimensions of MEMS resonators to achieve higher resonant frequencies, or immersing the resonator into liquid as a sensor [10,11,12,13], the parasitic capacitive feedthrough between the input and output ports becomes increasingly dominant, and the electrical path passing through the capacitor results in a significant drop in SBR (signal-to-background ratio). The above situation causes a tremendous challenge to full electrical characterization of resonators in micron scale (and even more severe for nano scale). This is because most signals generated by the resonator motion have been buried in background signals composed by electrical feedthrough. At the extreme limit, the SBR may decrease to a point where the resonant peak becomes undetectable [14,15].

Hence, exploring an appropriate solution to effectively cancel the parasitic capacitive feedthrough to significantly improve the SBR of the resonator in the context of full electrical characterization is highly required. One option to handle the problem caused by parasitic elements is to improve the fabrication process through monolithic integration of the MEMS and CMOS IC [16]. However, the successful development of a CMOS-MEMS process would be costly and restricted (e.g., due to type of material and thickness). The most popular approach to reducing feedthrough is by adopting a fully-differential configuration. Several capacitively transduced MEMS resonators using fully-differential configurations to overcome the feedthrough have been demonstrated [17,18]. For example, the parasitic capacitive feedthroughs were removed by exploiting the modulation of the transduction gap [19], a pseudo-differential method based on dummy resonators or dummy twin structures [20]. A series of other methods employing the combination of piezoresistive readout and capacitive input as a fully-differential configuration were also achieved [21,22,23]. These developed strategies provide a great opportunity to address the problem of parasitic capacitive feedthrough, but they make the implementations relatively complicated and their process costly. Furthermore, the main drawback of capacitive transduction is its low electromechanical coupling coefficient, which in turn leads to high motional impedance. Therefore, such a low electromechanical coupling coefficient brings with it a relatively high insertion loss.

In this paper, we propose a novel even-order width-extensional vibration TPoS MEMS resonator working in a fully-differential configuration to substantially suppress the undesirable electrical feedthrough posed by the presence of parasitic capacitive elements and to extract resonator parameters from raw measurements heavily buried in parasitic feedthrough. This on-chip solution presents a more compact size because only one single device is required. The proposed devices were fabricated by using a standard MEMSCAP process, which is suitable for mass fabrication and cost effective. The working principle of this unique configuration was systematically studied by both modeling simulation based on finite-element analysis and experimental comparison. The developed resonator successfully suppressed the feedthrough and achieved a higher SBR of 42.47 dB and lower insertion loss of 4.27 dB.

## 2. Resonator Model and Design

**Figure 1:**
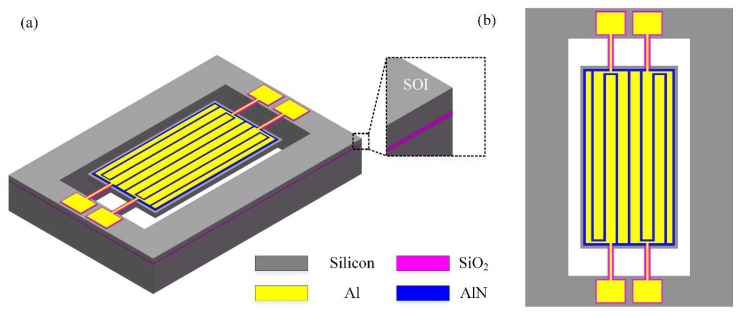
Schematic view of the designed thin-film piezoelectric-on-silicon (TPoS) resonator. (**a**) 3-D side view illustration of the resonator. The right inset image shows a high-magnification view of the SOI (i.e., silicon-on-insulator) substrate. (**b**) Vertical view of the resonator. This new design of the resonator operating in differential-in differential-out (DIDO) configuration reduces the insertion loss and suppresses the feedthrough.

Figure 1 shows the structure of the proposed differential input and differential output (called a fully-differential configuration) resonator. This four-port 8th-order width-extensional mode resonator was chosen as the test platform to study the effect of fully-differential configuration generated by even-order mode for parasitic capacitive feedthrough reduction. This device consists of a rectangular plate thin-film piezoelectric on silicon (TPoS) resonator body with a length of 300 µm and a width of 160 µm and two pairs of support tether beams. The resonator body consists of a piezoelectric layer sandwiched between a metal layer and a single crystal silicon layer. The piezoelectric sensing and driving are provided by this thin layer of aluminum nitride. The material of monocrystalline silicon was chosen to provide a high *Q* because of its low acoustic loss characteristics. Highly doped silicon is electrically conductive and is used as a ground electrode of this resonator. Four straight tether beams support the resonator body and provide an electrical path to the top electrodes. A vertical electric field applied across the piezoelectric film through the input electrodes results in a plane expansion of the resonant body through the d31 coefficient and consequently excites the resonator to vibrate in width-extensional mode. The output is sensed from the net charge, generated by the piezoelectric effect and induced at the electrode, then exports a signal.

**Figure 2:**
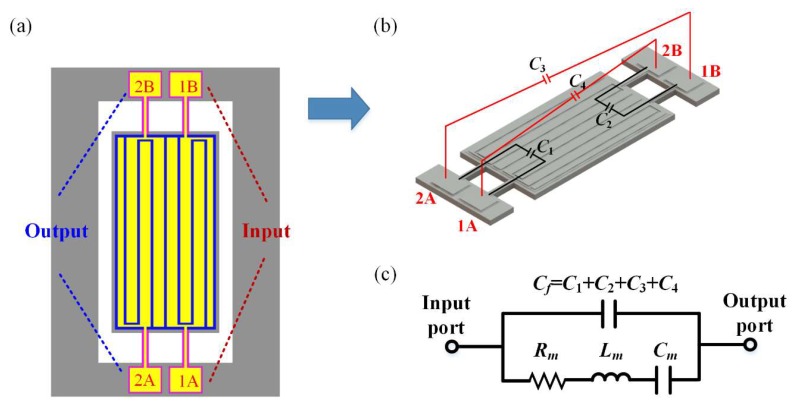
Schematic of the parasitic capacitive feedthrough in the proposed resonator. (**a**) Ports 1A and 1B are input ports, and ports 2A and 2B are output ports. The electrodes are divided into two sets: set A and set B. The electrodes in the same set are connected to input and output ports respectively (e.g., 1A and 2A). (**b**) *C*_1_ and *C*_2_ are the direct feedthrough between the input and output ports within one set. *C*_3_ and *C*_4_ are the cross feedthrough between the input and output ports belonging to different sets. (**c**) Traditional Butterworth-van Dyke (BVD) equivalent circuit model of the proposed resonator.

The configuration of the input and output ports of the resonator is shown in Figure 2a. The ports 1A and 1B are input ports, 2A and 2B are output ports. These ports are divided into two sets labeled A and B, respectively. Figure 2b shows the parasitic capacitive feedthrough in the proposed resonator. *C*_1_ and *C*_2_ are the direct feedthrough between the input and output ports within one set. *C*_3_ and *C*_4_ are the cross feedthrough between the input and output ports belonging to different sets. TPoS resonators can be represented by a lumped equivalent circuit model [24,25,26]. Considering that the input and output of the microelectromechanical resonator are electrical, the electrical equivalent circuit shown in Figure 2c is often used to describe the electromechanical characteristics of the resonator. In this BVD (Butterworth-Van Dyke) circuit model, the admittance network is composed of an *RLC* series resonant circuit and a shunt capacitor, *L_m_*, *C_m_*, and *R_m_* in this model represent the electromechanical dynamic characteristics of the device. This shunt capacitor *C_f_* acts as a parasitic feedthrough branch lying in parallel to the series resonant tank and is directly connected to the input and output of the device. The parasitic feedthrough capacitance includes capacitive coupling intrinsic to the resonator, such as direct coupling between the electrodes, coupling through the substrate, and external factors such as the interconnect wire and the package. The method proposed in this paper aims to suppress parasitic capacitances intrinsic to the device (i.e., *C*_1_, *C*_2_, *C*_3_ and *C*_4_).

**Figure 3:**
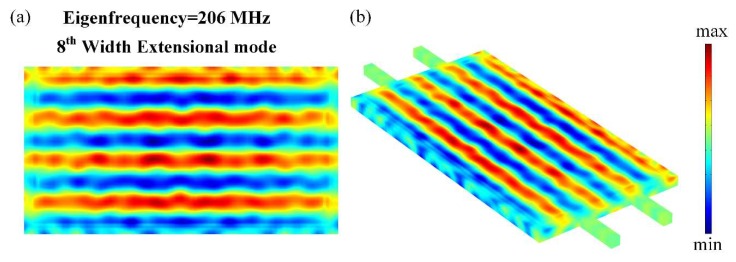
FEA (finite-element analysis) simulations were performed for the systematical investigation of the designed resonator. (**a**) is the displacement mode shape of the proposed resonator working in high order width-extensional (WE) vibration mode. It can be noticed from the displacement contour that the resonator was working in 8th-order width-extensional vibration mode and this fit the requirement of even-order. The support beams were positioned at the nodal points where the displacements were almost zero, as shown in (**b**).

The eigenmode of the proposed resonator was investigated based on FEA (Finite Element Analysis) by using COMSOL Multiphysics software shown in Figure 3. The resonant frequency of the width-extensional (WE) mode resonator was determined by the width of the resonator (2*L*) and the properties of the multilayer material (aluminum, aluminum nitride, and silicon) that make up the TPoS resonator. The high-order width-extensional modes excited and sensed by interdigitated transducer resembled by patterned top electrodes, can be determined by:

(1) $$f_{0}=\frac{n}{2L}\sqrt{\frac{E}{\rho }}$$

where *E* and *ρ* denote effective Young’s modulus and effective density of the stack of materials, respectively, and *n* is the order of resonance mode. As the order is increasing, the resonant frequency of the device also increases according to the formula.

Firstly, simulation of eigenmode was performed to determine the modality of interest. For operating in the differential-input differential-output (DIDO) configuration, the resonator must be designed to work in an even-order mode. Figure 3 shows the finite element simulation results of the displacement profile for the designed rectangular resonator operating in WE mode. With reference to Figure 3, the WE mode can be described as a rectangular plate contracting and extending along its width direction. It can be noted from the displacement contour that the expanded region and the contracted region are alternately arranged. The nodal location with a small displacement is located in the middle of the extension and contraction area. This resonator body is clamped by the suspended tether beams along the width of the plate and was designed to be operated in the 8th-order WE mode at 206 MHz, which is illustrated in Figure 3. Due to the Poisson effect in the silicon substrate, standing longitudinal waves in width are inevitably coupled to the length direction. Therefore, the tether beams were designed to be placed at the node points where the amount of displacement is minimum, as shown in Figure 3b. The location of the tether beam with minimum displacement can reduce the energy dissipated through the tether beams.

**Figure 4:**
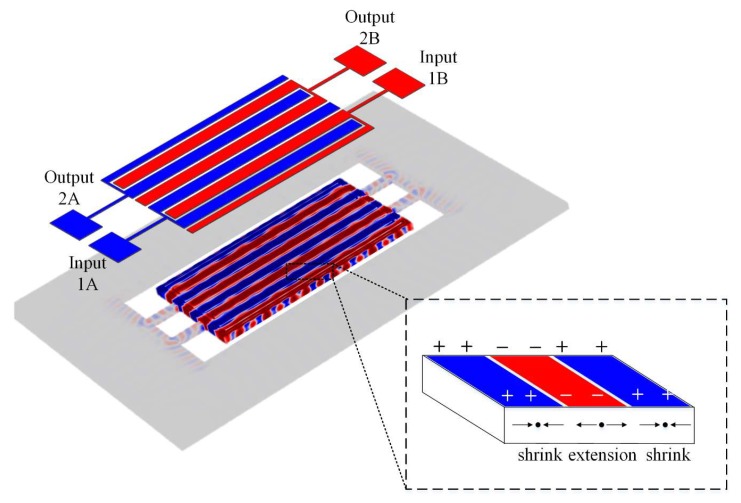
Illustration showing the volumetric strain profile of the 8th-order width-extensional vibration mode resonator. The distribution of strain polarities corresponds to that of displacement. The number of order is even. Insert provides the polarity and distribution of charge for this mode. Electrodes with the same amount were positioned on the corresponding strain regions. This DIDO configuration required a specific distribution of electrodes. All the electrodes were divided into two groups depending on the strain polarity of the region where they were positioned (i.e., group A and group B). So the polarities of these two groups were opposite. The electrodes on the same strain polarity regions were grouped together and then divided into input electrode and output electrode (e.g., 1A and 2A). Due to the specific distribution of these electrodes, when differential signals were applied to the input side, the proposed resonator operating in the DIDO configuration should output two anti-phase signals.

In order to correlate the charge generation of this structure with the displacement, it is necessary to analyze the resonant strain pattern of the resonator. Figure 4 illustrates the volumetric strain for the 8th-order width-extensional resonator of interest. It can be observed from Figure 4 that there are multiple distinct segments of strain for this even mode, and opposite strain region are separated from one another. According to the piezoelectric effect, the polarities of the charges generated on the surface of these different strain regions are also opposite.

To enable the resonator operating in a differential-in differential-out configuration (DIDO), special drive/sense electrode arrangements must be adopted. Eight top electrodes are sequentially distributed on the respective strain regions of the piezoelectric film. These eight top electrodes are divided into two groups depending on the polarity of the strain region. The strain regions in which each electrode in the same group is positioned have the same polarity. The in-phase top electrodes in each set of electrodes are interconnected to form a pair of driven electrodes and sense electrodes. One of the top electrodes is used to excite the resonator while the other top electrode is used to sense the motional current. It must be ensured that the driving electrodes in the two sets of top electrodes are out of phase and so as to the sense electrodes. The two sets of driving electrodes and sensing electrodes are correspondingly connected to the external port pads to form two input ports and two output ports of the resonator. The input signal is subjected to a differential input structure to generate two equal-power differential signals. These two signals are respectively applied into two driven electrodes on the strain regions, which are out of phase. Then two out-of-phase signals are generated through the two output ports, wherein the phases of these two output signals are mutually inverted. However, the feedthrough that reaches the sense electrodes through the driven electrodes directly is coupled to the output port through electrical coupling instead of generated by the mechanical vibration. Therefore, the feedthrough and input signals are in-phase. After the subtraction of these two output signals through the differential circuit, the output signal of the resonator is amplified because the resonant signals generated by the piezoelectric effect and sensed by the sensing electrodes are out of phase. The feedthrough is suppressed because two in-phase feedthrough signals from sensing electrodes cancel each other by subtraction. Therefore, the proposed fully-differential resonator can effectively suppress the feedthrough and improve the performance.

## 3. Results and Discussion

### 3.1. Fabrication

**Figure 5:**
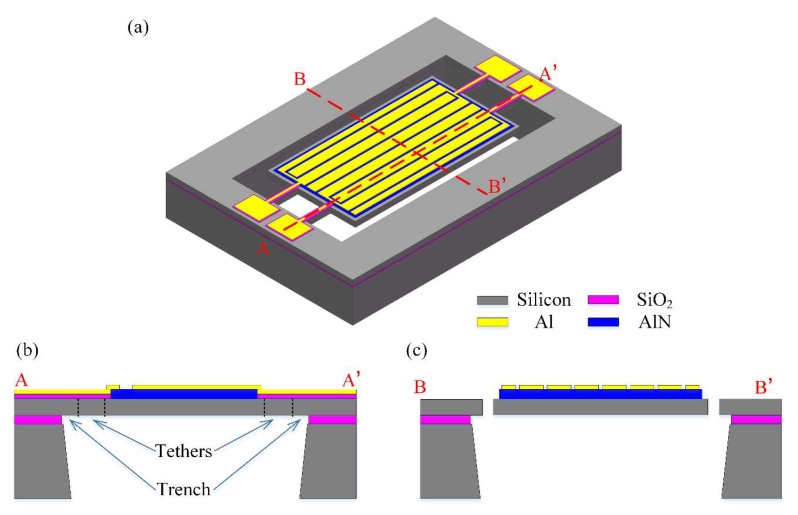
(**a**) The proposed resonator was fabricated using the MEMSCAP piezo micromachining process. (**b**) Illustrated cross-sectional schematic views indicated by the red dash line A–A’ show that there is a layer of oxide sandwiched between the metal transmission line and silicon layer for insulation. And the cross-sectional view schematic indicated by B–B’ illustrated in (**c**) shows more details about the electrodes and piezoelectric layer.

The designed even-order TPoS resonators were fabricated by using a foundry piezoelectric micromachining process through MEMSCAP. Figure 5 illustrates the cross-sectional schematic of the proposed fully-differential resonator with interdigital electrodes along A–A’ and B–B’. The total fabrication was a five masks process. In this fabrication process, a silicon-on-insulator (SOI) was used as the starting substrate (10 µm silicon, 1 µm oxide, 400 µm substrate). In this SOI wafer, the silicon layer was phosphorus doped as the ground electrode instead of a bottom metal electrode. Firstly, a thin thermal oxide layer (about 2000 Angstrom) was grown and patterned on the doped silicon layer. So the top metal electrodes at the tethers and anchor parts of the resonator were insulated from the doped silicon layer by this oxide layer rather than piezoelectric material. This treatment, replacing the piezoelectric material with the oxide layer, would avoid unwanted excitation at the tethers and help to further reduce anchor loss in turn. Then 500-nm thin film aluminum nitride (AlN) was deposited and patterned as the piezoelectric layer. A metal stack consisting of chrome (20 nm) and aluminum (1000 nm) was deposited by beam evaporation and patterned using lift-off process to define the pads, connecting wire, and top electrodes. The 10 µm silicon layer of SOI was etched using deep reaction ion etching (DRIE) to define the mechanical structure of the device. Finally, another DRIE process was used to etch the silicon substrate from the bottom side of the wafer to release the structure.

**Figure 6:**
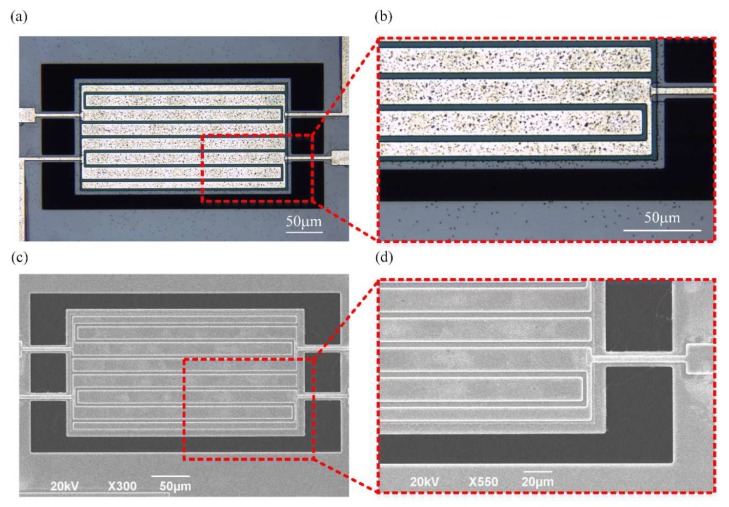
(**a**) The resonator with the DIDO configuration was fabricated using the MEMSCAP piezo micromachining process for the investigation of the effects of the DIDO configuration. High-magnification microscope images of (**b**) resonator with DIDO configuration exhibit the details of the resonator and also the interdigital electrodes. (**c**,**d**) shows the scanning electron micrographs of the resonator.

Figure 6a shows the top view of microscope images of the fabricated 8th-order width-extensional mode TPoS resonators. The high-magnification microscope image of the interdigital electrodes section of the resonator is given by Figure 6b. The fabricated resonator was hermetically sealed in a ceramic leaded chip carrier in barometric pressure. The chip and pads on the carrier were connected using gold wire bonding, and the lid was bonded to the carrier using epoxy resin to protect the device from pollution. The feedthrough of packaged resonator was larger than that of the bare die. The feedthrough was artificially introduced to the device.

### 3.2. Measurement and Results

**Figure 7:**
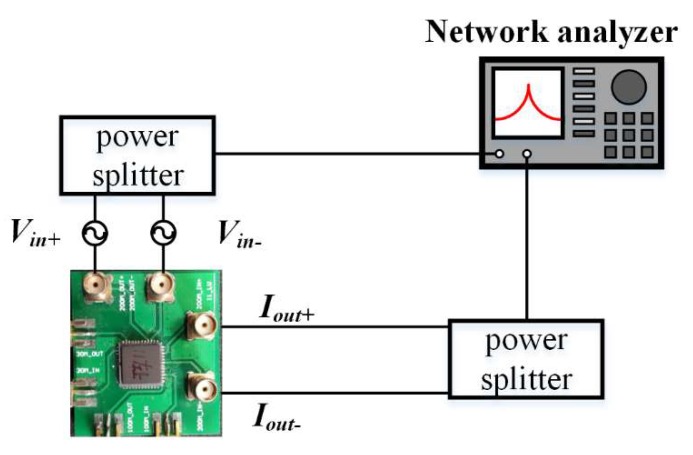
Measurements of *S*-parameters of the fabricated TPoS resonators in a four-port DIDO configuration. Schematic diagram of the electric measurement platform. The proposed resonator was connected to a network analyzer to measure its transmission *S*_21_ at the resonant frequency of 206 MHz with a 20 MHz span.

A circuit schematic for the width-extensional mode measurement setup is described in Figure 7, which shows a fully-differential configuration to suppress feedthrough. The measurement of electrical transmission of this resonator was done using an R&S vector network analyzer (ZVL 6) after short-open-load-through calibration. Input signals were fed to the two sets of input electrodes, while on the other two sets of electrodes output signals were sensed.

**Figure 8:**
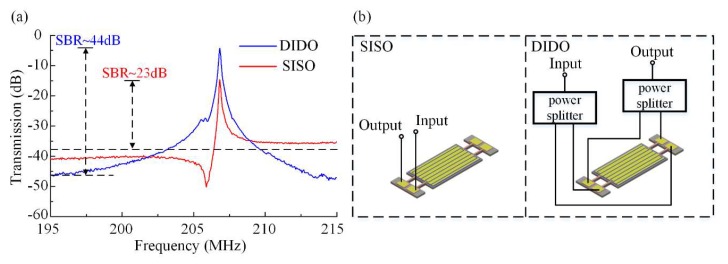
Measurements of *S*-parameters of the fabricated TPoS resonators in differential configurations. (**a**) The measured response of the single terminal in single terminal out setup (SISO) is shown as the red trace, and the blue trace is the transmission plot for the DIDO setup. A resonant peak with high signal to background ratio (SBR) is achieved by the DIDO setup. The SBR of SISO is much lower than DIDO because of no cancellation of the common-mode feedthrough signal. The insert loss of the DIDO setup is much better than the SISO setup because this setup of DIDO can enhance the differential-mode signal. (**b**) Measurement configurations of resonator operating in SISO and DIDO.

The input and output setup used in this work included several different configurations designed to work at the same frequency. A confirmatory measurement procedure was performed by taking into account these different configurations of ports. We measured the electrical transmission *S*_21_ of the proposed device first using the single terminal in single terminal out (SISO) configuration (without feedthrough cancellation), then the proposed feedthrough cancelling configuration of differential-in differential-out (DIDO), followed by two configurations for comparison, single terminal in differential-out (SIDO) and differential-in single terminal out (DISO), respectively. The common-in common-out configuration was not used here because the common-mode signals fed on the regions with strains of opposite polarity will cancel each other. The features of different configurations were made use of to confirm the effectiveness on feedthrough cancellation. The results of SISO as a pristine reference and the proposed DIDO are shown in Figure 8.

Two out-of-phase input signals were fed into the resonator and two output signals were differentially processed. This setup of terminals was chosen to constitute the DIDO configuration. The blue trace in Figure 8 is the transmission characteristic measurement result for the DIDO configuration. A resonant peak with low insertion loss and high SBR was achieved. In this configuration the insertion loss was about 4 dB and the SBR was about 42.47 dB. The opposite polarity strains of the regions on which the two sets of sense electrodes were located, combined with the outside differential setup, made the resonant peak very strong, and at the same time it canceled out the common-mode signal, i.e., the parasitic capacitive feedthrough. In the SISO configuration, the input signal was fed to one of the input port and one of the sensed signal was directly output. The measured response of the DIDO configuration is shown as the blue trace. The insertion loss in the SISO configuration was 14.65 dB, and SBR was only 26.56 dB, 15.91 dB lower than DIDO. In this configuration, the SBR was much lower than that of the DIDO configuration because there was no cancellation of the common-mode signal. Hence the parasitic capacitive feedthrough as the common-mode signal was retained. Simultaneously, one sensing signal without differential process led to a relatively low resonant peak and the insertion loss was unsatisfying.

### 3.3. Discussion

**Figure 9:**
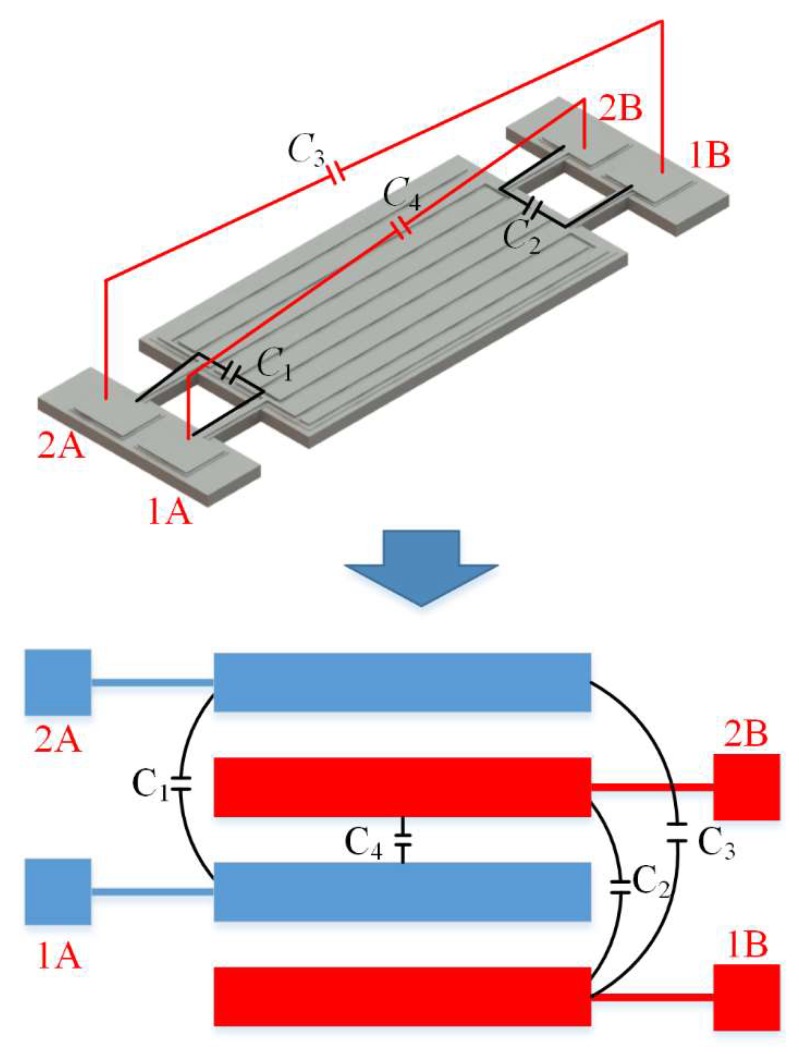
Schematic of the parasitic capacitive feedthrough in the proposed resonator. These ports are divided into two sets. The electrodes in the same set connected to input and output ports respectively are located on the regions with same strain polarity. The polarity of electrodes of these two sets of ports are inverse. *C*_1_ and *C*_2_ are the direct feedthrough between the input and output ports within one set. *C*_3_ and *C*_4_ are the cross feedthrough between the input and output ports belonging to different sets.

The results presented above show that the performance of the resonator in the DIDO configuration was much better than in SISO. It should be pointed out that the degree of successful feedthrough cancellation depended on the configuration of the input and output of the proposed resonator. In this section, we demonstrate the results obtained using different configurations to illustrate the physical basis of the observed parasitic capacitive feedthrough cancellation. In order to achieve a balanced input, the differential inputs need to be applied to an equal number of electrodes, so as to the output. Four sets of electrodes are used in total. Two sets of them positioned on the same polarity strain regions are connected to the input 1A and output 2A respectively and the remaining two sets on the regions where strain polarity is opposite to previous sets connect the input 1B and output 2B. Figure 4 presents the strain distribution of the proposed resonator. On the resonator body, regions with opposite strain polarity are arranged alternately and the polarities of charges on these regions are opposite. The interval distribution of the electrical characteristics is utilized for electrode arrangement. The two pairs of driving/sensing electrodes are located on opposite polarity regions, causing the electrical characteristics of the two output ports to be inverting. This mechanism of invert output signals makes the resonator a differential device, so as to suppress parasitic capacitive feedthrough and spurs. The parasitic capacitive feedthrough in the proposed device is shown in Figure 9. In the following sections, the electrical characteristics of the resonator applied in different configurations are studied in detail.

For further explanation, the interdigital electrodes of the resonator can be obtained in an equivalent simplified form to describe the feedthrough capacitance as shown in Figure 9. In this figure, ports 1A and 1B are input ports, 2A and 2B are output ports. Parasitic feedthrough capacitances exist between the respective input and output ports. In Figure 9, *C*_1_ is the feedthrough capacitance between the first pair of in-phase ports (i.e., input port 1A and output port 2A). *C*_2_ is the feedthrough capacitance between the second pair of in-phase ports (i.e., input port 1B and output port 2B). *C*_3_ and *C*_4_ are the cross-feedthrough capacitances between the input and output ports from the two pairs of ports. It will be observed that the configurations of input and output ports affect the degree of feedthrough cancellation and insertion loss. Three different setups are used to verify the effects of feedthrough cancellation by the differential configuration.

**Figure 10:**
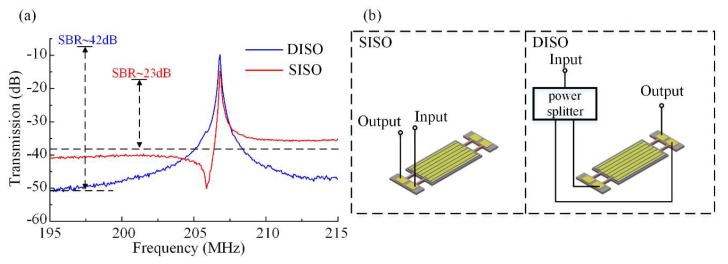
Comparison of the electrical transmission of the proposed resonator working in SISO and DISO setups. (**a**) The red trace is the results for SISO, and the response of the DISO setup is shown as the blue trace. Measurement result for DISO is better than that for SISO in both insertion loss and feedthrough suppression. (**b**) Measurement configurations of resonator operating in SISO and DISO.

In the first configuration, two out-of-phase input signals were fed into the resonator and one of the sensed signals was directly output. This setup of terminals was chosen to constitute the DISO configuration. We characterized the resonator using a different input configuration, first combing both input ports differentially, followed by feeding in just one of the input ports (SISO). The blue line in Figure 10 is the measurement result for the DISO setup, and the transmission characteristic of SISO is expressed by the red trace. According to Figure 10, with a DISO setup, the measured feedthrough transmission is −51.56 dB, and the insertion loss is 9.78 dB. When using the SISO configuration, the effect of feedthrough capacitance is so great that the feedthrough is only −41.21 dB. The SBR of 41.78 dB for DISO is much better than that of the SISO configuration by about 15.22 dB. In the configuration of SISO, the feedthrough from input port 1A/1B is transmitted to output port 2A/2B directly. When the resonator is working in the DISO configuration, the feedthrough from input ports 1A and 1B to 2A can cancel each other because these two input signals are out of phase. The same happened to the feedthrough between input ports 1A and 1B to output port 2B. These results suggest that the parasitic capacitive feedthrough of the resonator is suppressed sharply by this differential input configuration.

**Figure 11:**
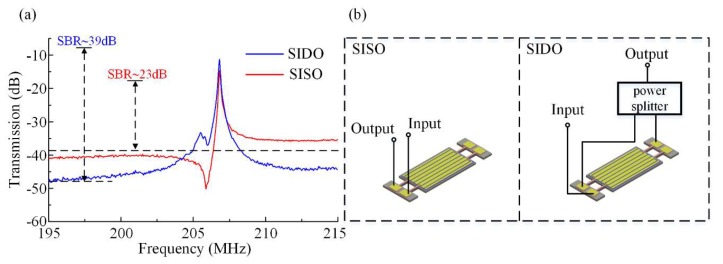
Measured electric transmission for a resonator operating in SIDO (blue trace) and SISO (red trace) setups. (**a**) The red trace is the result for SISO, and the response of the SIDO setup is shown as the blue trace. Measurement result for SIDO is better than that for SISO in both insertion loss and feedthrough suppression. (**b**) Measurement configurations of resonator operating in SISO and SIDO respectively.

In the SIDO configuration, one input signal is fed to one of the input ports, and two out-of-phase output signals are obtained through the resonator. These two signals combine in a differential setup to subsequently obtain a final output signal. As illustrated in Figure 11, the measured response of the SIDO setup is compared with that of the SISO setup. The blue trace in Figure 11 is the result for SIDO and the red one is for SISO. Comparing the measurement results of SIDO against that of the SISO configuration, we can see that feedthrough was lowered substantially by 7.67 dB. These results indicate that the differential output configuration also affects the level of feedthrough cancellation of the resonator. In the SIDO configuration, two output signals combine in a differential setup. So the common-mode signals in these two outputs cancel each other out. As such, feedthrough as the common-mode signal will be suppressed in this configuration.

**Figure 12:**
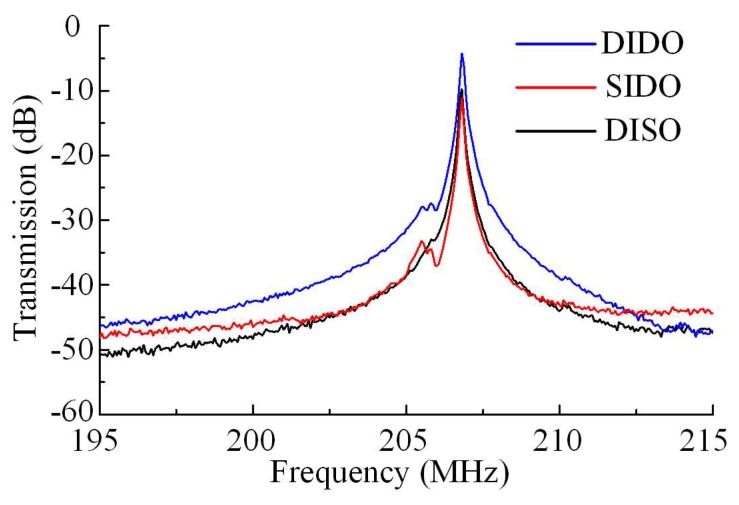
Measured electromechanical frequency response to compare the effect of using the DIDO setup against the SIDO and DISO setups. The feedthrough suppression measured in the SIDO setup is slightly different from that in DISO setup. Both of them suppress the feedthrough better than the DIDO setup, but the insertion loss measured in the DIDO setup is the best.

In conclusion, we compared all the four configurations (i.e., SISO, DISO, SIDO, and DIDO). Measured results of the resonator working in all these configurations are compared in Figure 12. SISO without any differential configuration was the basic setup of the resonator, and served as a benchmark for evaluation. From the measured electrical transmissions shown in Figure 12, all of the setups with differential configuration achieved better performance than SISO. They got lower insertion loss, smaller feedthrough and better SBR. The feedthrough in each instance was different from each other. Although different differential configuration setups were used in all these three instances, the disparity of resulting feedthrough cancellation among them was not so huge. Furthermore, the feedthrough cancellation of the setup with the fully-differential configuration was not the best in these instances. This comparison suggests that different differential setups have different effects on feedthrough suppression.

The observations can be explained with reference to Figure 9. As illustrated in Figure 9, the net parasitic feedthrough of the resonator was *C*_1_/*C*_2_ in the SISO configuration. The feedthrough had a substantial influence on the electrical characteristics. In the DISO setup, the input signal split into two inverting paths, one to each input port. So feedthroughs from each input port were fed to the unique output port directly. These feedthroughs were out of phase relative to each other. The net feedthrough in this configuration was about *C*_1_–*C*_3_ or *C*_2_–*C*_4_, depending on the choice of output port. In the SIDO setup, only one input signal was fed to the resonator. Part of the input signal was fed to each output port. The feedthroughs sensed from output ports were in-phase. After combining outputs differentially, the net feedthrough was about *C*_1_–*C*_4_ or *C*_3_–*C*_2_, considering the feedthrough pathways in Figure 9. Last of all, in the case of the DIDO setup, a fully-differential setup was adopted to achieve the objective of feedthrough cancellation. The resonator working in the DIDO setup possessed two input paths which were equal in magnitude but out of phase relative to each other. Feedthroughs from each input port were fed directly to the two output ports. And the output ports of the resonator were combined differentially. So the feedthrough readout at output ports were combined differentially to get the net feedthrough, about *C*_1_–*C*_3_ + *C*_2_–*C*_4_. That caused a larger feedthrough of DIDO than that of SIDO and DISO. Despite this, the feedthrough suppression of DIDO was enhanced. In addition, the setups with differential-out configuration enhanced the differential-mode signals, i.e., resonant signals here. So the insertion loss was improved.

## 4. Conclusions

A unique design of a 206.8 MHz TPoS MEMS resonator working in even-order width-extensional vibration mode was proposed to cancel the parasitic capacitive feedthrough in this paper. We used the fully-differential configuration that has commonly been applied to a capacitive resonator on the TPoS resonator to enhance the SBR in the context of full electrical characterization. This designed feedthrough cancellation solution combines the merits of strong electromechanical coupling from TPoS technology with the merits of common-mode rejection from a fully-differential configuration. The systematical investigations, including FEA simulations and experimental comparison, confirmed the feedthrough cancellation via the improvement of SBR. The highest value of SBR measured for the proposed device operating in the DIDO configuration was found to be 42.47 dB, which is about 1.6 times larger than that of the resonator working in the SISO configuration. And the insert loss was also improved from 14.65 dB to 4.27 dB. This suggests that the combination of fully-differential configuration with TPoS appears to be effective for enhancement of SBR by suppressing the feedthrough. We anticipate that the proposed solution could open up a new avenue in the design of MEMS resonator devices where the parasitic capacitive feedthrough issues can be addressed effectively and at low cost.
